# Polarization-insensitive Archimedes’-spiral-shaped ultrathin metamaterial absorbers for microwave sensing application

**DOI:** 10.1038/s41598-023-46363-x

**Published:** 2023-11-09

**Authors:** Omar S. Lateef, Mohammed Al-Badri, Khalid Saeed Lateef Al-badri, Sarah Adnan Mohammed

**Affiliations:** 1 Chemical Engineering Department, Tikrit University, Tikrit, Salah al deen Iraq; 2 Physics Department, University of Samarra, Samarra, Salah al deen Iraq; 3 Electrical Engineering Department, University of Samarra, Samarra, Salah al deen Iraq

**Keywords:** Engineering, Materials science, Optics and photonics, Physics

## Abstract

This work has developed and simulated a planar complementary Archimedes-based metamaterial absorber with the goal of its application in refractive index sensing. Unlike designs that employ multiple layers or numerous resonators within a single unit cell, our proposed absorber adopts a more streamlined approach. It consists of three layers, with an FR4 dielectric substrate sandwiched between two copper layers. It's important to note that the absorption characteristics of this design are polarization-dependent. This polarization dependence arises from the asymmetrical resonance behavior observed in both the x and y directions. The absorber exhibits impressive absorption rates at various resonance frequencies, namely *98.5*% at *f*_*1*_ = *8.49 GHz, 77.1*%* at f*_*2*_ = *8.88 GHz, 88.7*%* at f*_*3*_ = *9.3 GHz, 98.2*%* at f*_*4*_ = *9.87 GHz, 99.7*%* at f*_*5*_ = *10.65 GHz, 83.4*%* at f*_*6*_ = *11.58 GHz, and 99.9*%* at f*_*7*_ = *12.24* GHz. Furthermore, the article explored the refractive index sensing capabilities of this structure by introducing a 1 mm analyte layer on top of the patch structure. Through refractive index sensing analysis, we've determined that this absorber-based sensor yields an impressive high-quality factor value of 84.5, highlighting its remarkable sensitivity and precision. A more profound comprehension of the physical mechanisms in action has been attained by examining the distribution of surface currents. Furthermore, the behavior of the absorber has been investigated under varying polarization and incident angle conditions, ranging from zero degrees to sixty degrees. The thorough characterization establishes this absorber as a promising choice for microwave sensing applications.

## Introduction

Since the groundbreaking work of Landy et al.^1^ on perfect metamaterial absorbers (PMMA), this concept has gained significant attention in the field of science and technology. PMMAs have been extensively developed across various electromagnetic (EM) spectrum ranges, including microwave^1–3^, terahertz^4–11^, infrared^12–16^, and visible regions^17–20^. The advantage of PMMA surface current distribution lies in its geometric scalability, allowing it to operate at different EM spectrum ranges without being limited by the quarter-wavelength thickness. PMMAs have found applications in thermal imaging^12^, sensors^16,21–23^, solar cells^19^, thermal emitters^24^, and more. Typically, a PMMA consists of three layers: a patterned metallic structure serving as the EM resonator, a dielectric or magnetic spacer, and a continuous metal film or metal wire as the ground layer. By adjusting the geometric parameters and properties of these layers, the PMMA can achieve near-unity absorption based on the fundamental resonance of the EM resonator.

Efforts have been made to achieve multi-band or broadband high-level absorption for EM waves^25–32^. Two design strategies have been employed: combining multiple sub-units within a super-unit resonant structure^6–8^, or constructing alternating patterned metallic structures and dielectric layers with different geometric parameters^27–31^. However, both strategies have limitations, such as increased fabrication cost, angular dependence, and neglecting high-order EM responses. High-order resonances of metamaterials (MMs) are often overlooked in PMMA designs, but they can play a crucial role in achieving multi-band absorption. Several studies have demonstrated the combination of fundamental and high-order resonance modes in a single patterned metallic structure^3,8,11,33–35^, resulting in multi-band absorption.

Metamaterial absorbers are predominantly reliant on their robust resonance characteristics, which offer a significant advantage. These sensors can generate a powerful and easily measurable signal through a resonance absorption peak. This signal is potent enough to accurately monitor shifts in absorption spectra^2,3,17,21^. Essentially, when alterations occur in the dielectric substrate or geometric structure due to external influences, the electromagnetic properties of the metamaterial change accordingly. This leads to variations in resonance frequency and absorption intensity. If there's a direct and proportional relationship between these frequency or absorption changes and external influences, metamaterial absorbers can effectively identify changes in those influences. This versatility extends to parameters like dielectric constants, substrate thickness, biological features, and more^2,22,31^.

However, it’s important to note that research on the sensing applications of metamaterials has predominantly concentrated on the terahertz and infrared bands^7,18,31^. Conversely, investigations into the microwave waveband have lagged somewhat behind, limiting their broader applicability. Moreover, many microwave and metamaterial absorber-based sensors often necessitate specific samples that have undergone destructive treatments or involve liquid samples. This requirement is due to their restricted sensing areas, which also diminishes their suitability for non-destructive sensing applications.

This paper presents a simple and effective design of an ultrathin seven-band polarization-sensitive PMMA in the X-band microwave region. The design consists of an array of Complementary Archimedes Resonator CAR and a copper ground plane separated by a thin FR4 dielectric film. The PMMA exhibits seven narrow absorption bands with resonance peaks exceeding 77.1–99.9%. Compared to previous PMMA designs, our approach offers compact unit size, novel resonance mechanism, polarization sensitivity, and higher Q factors. The simplicity and effectiveness of this design hold potential for applications in biological sensing, material detection, thermal imaging, and microwave communications.

## Design

**Figure 1:**
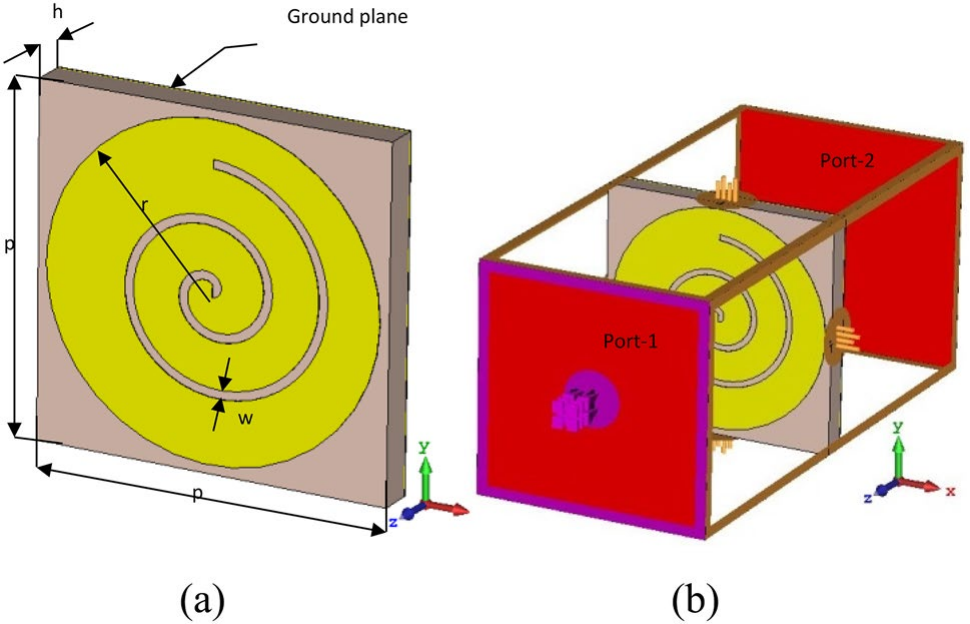
(**a**) Configuration of the proposed unit cell, (**b**) CST simulation setup.

The artificial metamaterials absorption characteristic depends on some factors including the electromagnetic properties of dielectric substrate and structure topology of the unit-cell. In this project, the substrate is FR4 with permittivity *ε*_*r*_ = *4.6*, loss tangent *tan δ* = *0.015,* and thickness *h* = *2* mm. The front face of substrate is planar complementary archimedes based on patch circle. And the back side is metallic plane. The metallic layers are copper with a thickness is *t*_*c*_ = *35 µm* and electrical conductivity constant is *σ* = *5.8* × *10*^7^ S/m. The structure parameters are listed in Table 1 and labelled in Fig. 1a. Where the archimedes is designed according to Eqs. (1) and (2).

1 $$x(t)=g\times t \times \cos (t),$$

2 $$y(t)=g\times t \times \sin (t),$$

where $$0\le t\le n\pi$$.

**Table 1:** Structure parameters.

Parameter	Value (mm)
*p*	20
*r*	9.5
*h*	2
*w*	0.5
Other parameters: *g* = 0.5 and *n* = 4.5

The simulations have been performed by using computer simulation technology (CST) Microwave Studio commercial software, which is based on frequency domain solver and finite element analysis. The boundary condition of unit cell is employed to simulate the infinite periodic structure in x- and y-directions, and open (add space) boundary is used in z-direction. The absorber is excited by an incident plane wave along the negative direction of the z-axis by using the Floquet port. Figure 1b shows the simulation setup when the electromagnetic propagation vector is perpendicular to the cross-section of the PMMA structure i.e. along z direction. The electric field and magnetic field are applied along y and x direction respectively. The two-dimension scattering parameters are obtained by CST in order to calculate the frequency dependent absorption spectrum $A(\omega )$ according to Eq. (3).

3 $$A(\omega )=1-R(\omega )-T(\omega ),$$

where $R(\omega )={|{S}_{11}|}^{2}$ is the reflection coefficient, and $T(\omega )={|{S}_{21}|}^{2}$ is the transmission coefficient. The back side of substrate is fully covered by the copper layer which prevents wave from passing through structure, therefore $T(\omega )=0$. Thus, the perfect absorption occurs when zero reflection.

## Results and discussion

**Figure 2:**
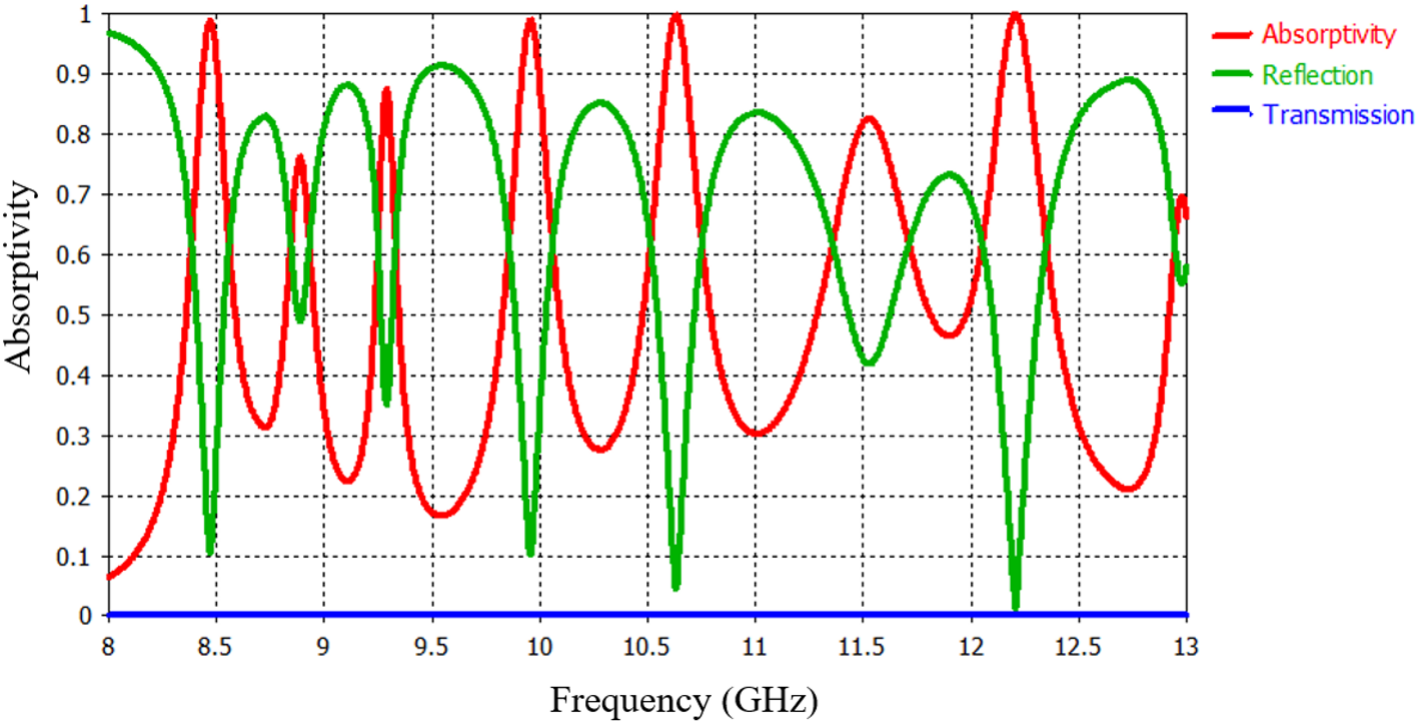
S-parameters and absorptivity of the proposed PMMA.

The results and discussion of the proposed PMMA are presented in this section. Figure 2 displays the simulated absorbance spectra of the PMMA, clearly showing seven resonant frequencies (*f*_1_*, f*_2_*, f*_3_*, f*_4_*, f*_5_*, f*_6_ and *f*_7_). At these resonant frequencies (*f*_*1*_ = *8.49* GHz, *f*_*2*_ = *8.88* GHz*, f*_*3*_ = *9.3* GHz, *f*_*4*_ = *9.87* GHz, *f*_*5*_ = *10.65* GHz, *f*_*6*_ = *11.58* GHz and *f*_*7*_ = *12.24* GHz), the absorbance A(ω) is approximately *98.5*%*, 77.1*%*, 88.7*%*, 98.2*%*, 99.7*%*, 83.4*% and *99.9*%*,* respectively (Fig. 4b–g). The corresponding electric thickness of the PMMA relative to the resonance wavelength (λ_i_) is approximately *0.056λ*_*1*_*, 0.059λ*_*2*_*, 0.062λ*_*3*_*, 0.066λ*_*4*_*, 0.071λ*_*5*_*, 0.077λ*_*6*_ and *0.082λ*_*7*_, respectively. Thus, the designed PMMA exhibits an ultrathin thickness compared to the operating wavelength. The PMMA demonstrates frequency selectivity, with narrow bandwidths of perfect absorption. The high-level absorption at each resonant frequency is attributed to the dipole resonances of the CAR structure. The absorption frequency band of the seven-peak PMMA is relatively narrow compared to previous PMMAs. This design is expected to have significantly higher Q factors than previous designs. The Q factor, defined as the ratio of the central frequency to the full width at half maximum (FWHM) bandwidth, is calculated for each resonance. Consequently, the corresponding Q factors are approximately *Q*_*1*_ = *41.2, Q*_*2*_ = *80.73, Q*_*3*_ = *84.5, Q*_*4*_ = *38, Q*_*5*_ = *35.3, Q*_*6*_ = *13* and *Q*_*7*_ = *30.6*. These results indicate that the high-level absorption with high Q factors occurs only at resonant frequencies. Compared to previous MM structures used in sensing applications (*Q factor* < *20*), the proposed PMMA exhibits relatively higher Q factors, making it highly suitable for sensitive sensing applications such as phase imaging of prohibited drugs, detection of combustible, toxic, and harmful gases, and biological sensing. Additionally, the proposed PMMA structure is expected to be sensitive to the polarization state of incident waves due to its geometric rotational asymmetry.

**Figure 3:**
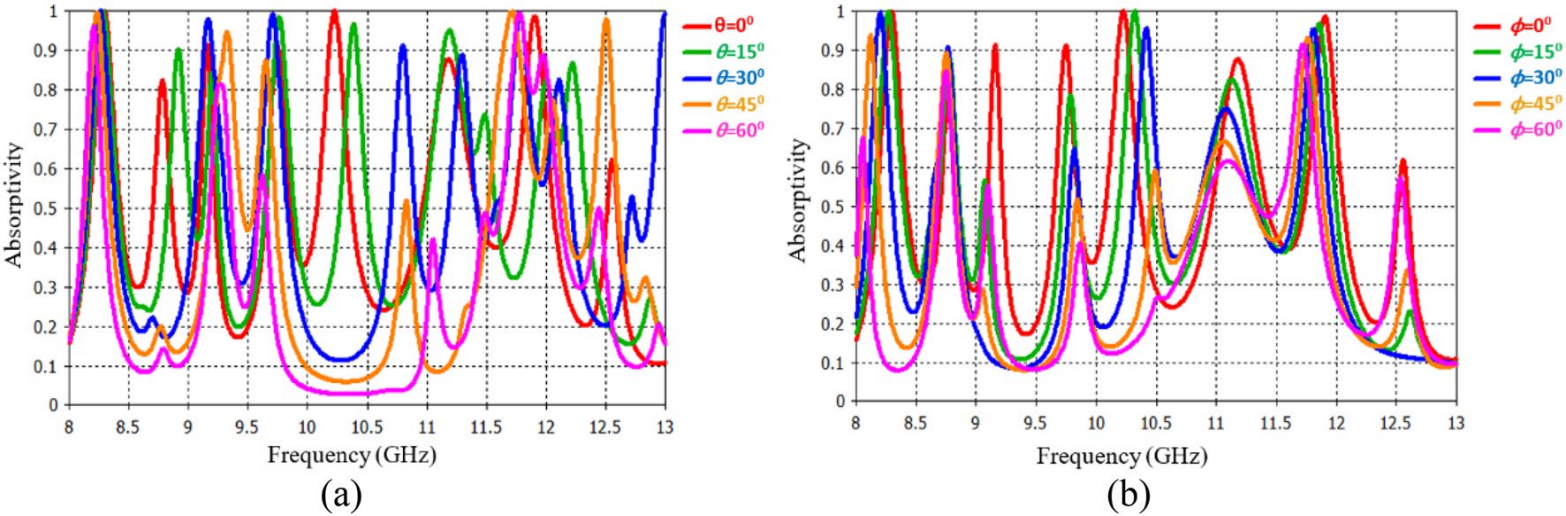
The TE/TM absorption response under normal incidence (**a**) TE mode and (**b**) TM mode of operation.

The polarization angle dependence of the PMMA was assessed for TE and TM waves under normal incidence, as depicted in Fig. 3a and b. The unit-cell structure of the PMMA possesses rotational asymmetry, it has been examined the absorption of PMMA for five TE/TM incidence angles (*0*°*, 15*°, *30*°, *45*° and *60*°). The findings indicate that when waves under normal incidence, absorbance do not remain constant across different polarization angles in both TE and TM modes. This indicates that PMMA exhibits polarization sensitivity, making it suitable for polarization-independent applications. Notably, the absorbance for the first and seven resonances remains steady for both TE and TM modes when the incident wave angle is below *60*°. However, as the incident angle increases (θ > 45°), the performance of higher-resonant frequencies deteriorates due to the impact of higher-order multipolar plasmon resonance.

**Figure 4:**
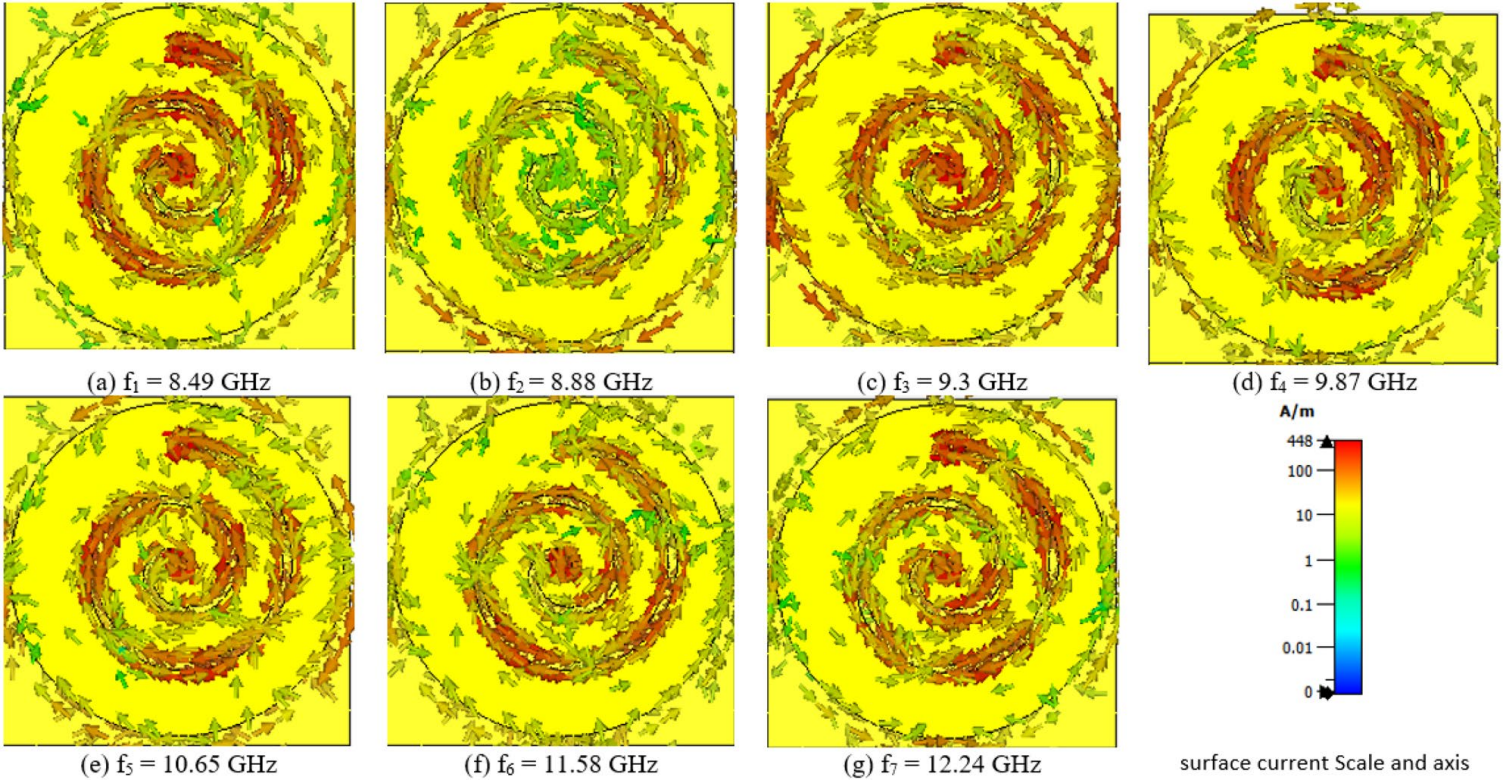
Surface current distribution across seven resonance peaks. (**a**) 8.49 GHz, (**b**) 8.88 GHz, (**c**) 9.3 GHz, (**d**) 9.87 GHz, (**e**) 10.65 GHz, (**f**) 11.58 GHz and (**g**) 12.24 GHz.

In order to gain a comprehensive understanding of the physical mechanisms underpinning the seven-band absorptions, an examination of the current distribution on the metallic structures, as depicted in Fig. 4b–g, was undertaken. In this figure, directional information is denoted by arrows, while intensity is conveyed through color gradients. Analysis of this figure reveals the presence of seven distinct resonant modes that are notably discernible within the surface current distribution. Specifically, the first, second, and fourth resonant frequencies at 8.49 GHz, 8.88 GHz, and 9.87 GHz exhibit polarization in the Z plane (Fig. 4a,b and d respectively). These modes manifest an anticlockwise direction during the first and second laps, followed by a clockwise direction in the third lap. Conversely, the third resonant frequency at 9.3 GHz demonstrates Z-plane polarization, with clockwise direction during the initial two laps and an anticlockwise direction in the third lap Fig. 4c. The fifth resonant frequency Fig. 4e, positioned at 10.65 GHz, showcases Z-plane polarization, following a clockwise direction in the first lap and an anticlockwise direction in the second lap. At the sixth resonant frequency of 11.58 GHz Fig. 4f, Z-plane polarization is again observed, with a clockwise direction spanning the second and third laps. Finally, the seventh resonant frequency Fig. 4g, at 12.24 GHz, features Z-plane polarization with an anticlockwise direction in the first lap and a clockwise direction in the third lap. This comprehensive analysis elucidates the distinct polarization characteristics and directional behavior associated with each of the seven resonant frequencies.

### Parametric analysis

This section presents a comprehensive parametric investigation aimed to understand the behavior of the proposed structure. During the design phase, the Archimedes structure's performance is affected by changes in key factors like the Archimedes resonator width, the number of loops, and the substrate thickness.

**Figure 5:**
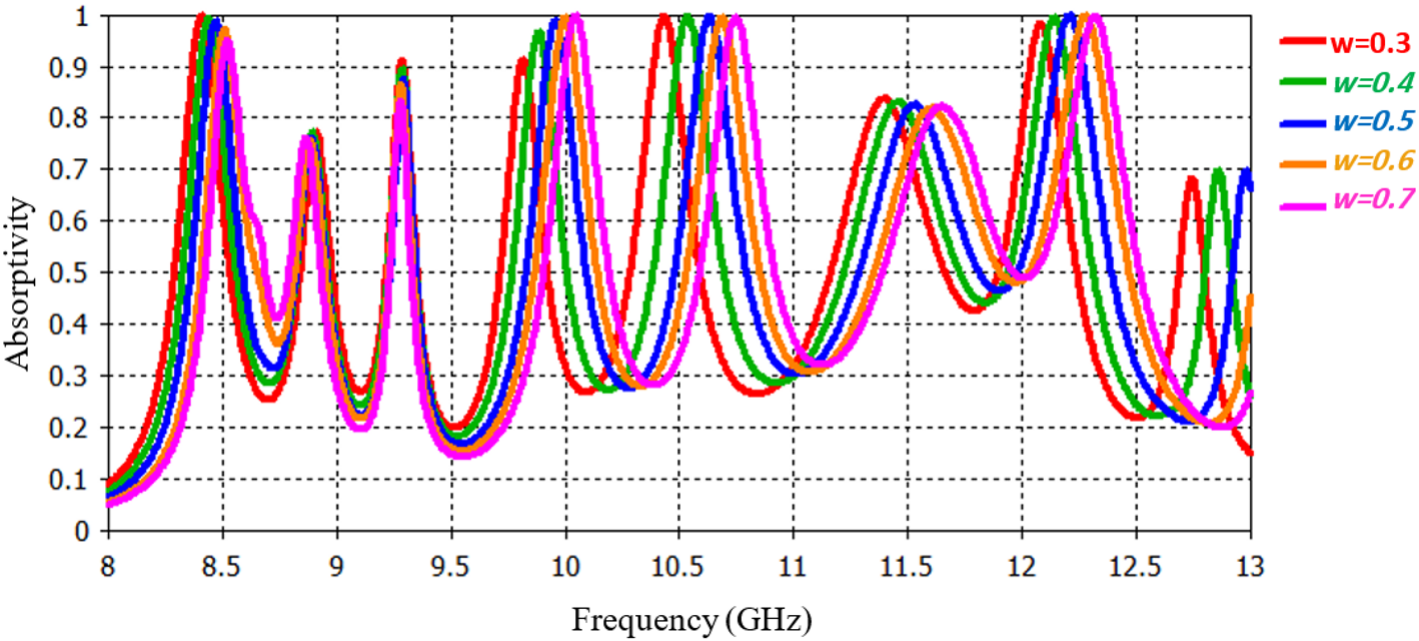
Absorption response for various width of the resonator Archimedes.

In particular, it has been focused on the analysis of the Archimedes width, as it plays a pivotal role in shaping the behavior of the metamaterial Resonator. Also, it has been examined the reflection coefficient (S_11_) of PMMA for five distinct width values (*w* = *0.3 mm, 0.4 mm, 0.5 mm, 0.6 mm, and 0.7 mm*). Figure 5 visually depicts the reflection characteristics corresponding to these different w values. The analysis shows that resonance frequencies are directly proportional to w values. An increase in w values results in higher resonance frequencies, while a decrease in w width leads to a reduction in the capacitance of the resonator, consequently amplifying the resonances observed in the reflection spectrum. Among the various w values assessed, a width of 0.5 mm demonstrates superior performance, and as such, we have selected it for the design of our proposed structure.

**Figure 6:**
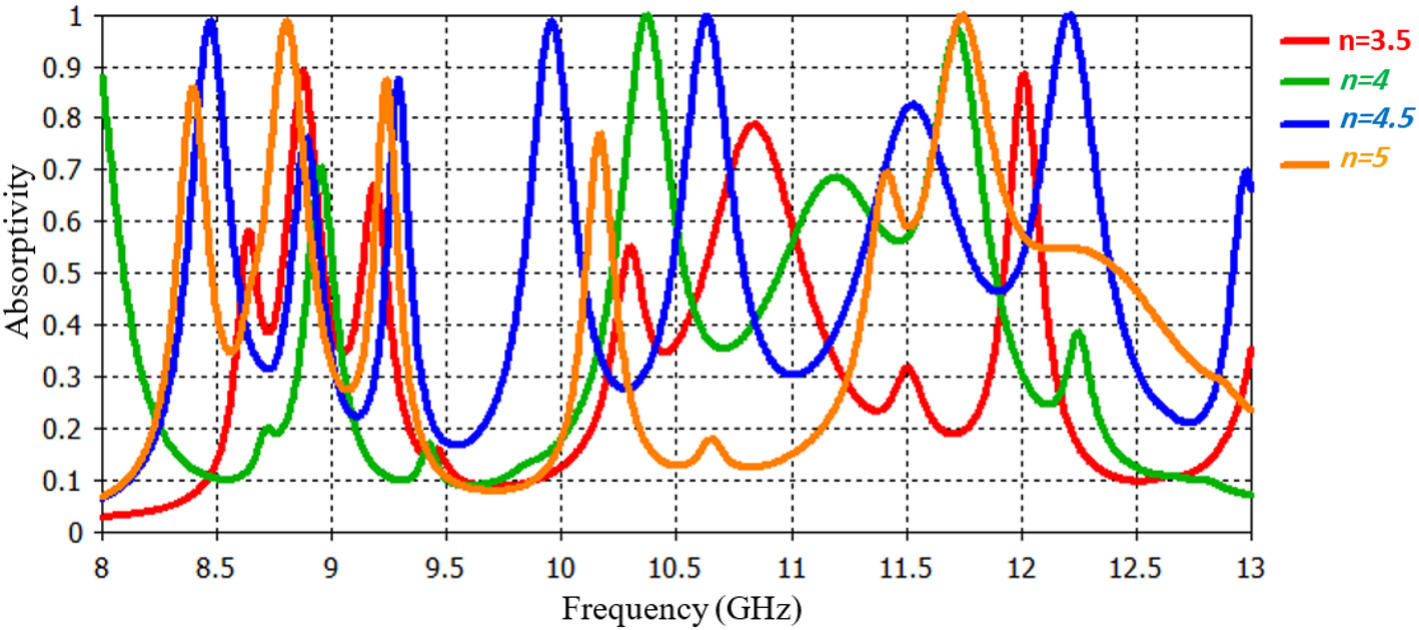
Absorption response for various number of Archimedes loops.

The variable representing the number of Archimedes loops, denoted as ‘n’ was systematically varied within the range of n = 3.5 to n = 5, with an incremental step size of 0.5. Interestingly, when ‘n’ is set to 4.5, the absorber shows remarkable resonance behavior. It absorbs electromagnetic waves at seven different frequencies with much higher rates compared to other ‘n’ values. Figure 6 represents absorption rates at various frequencies, highlighting the resonance peaks at 'n' equals 4.5. This finding underscores the significance of the ‘n’ parameter in shaping the absorptive properties of the Archimedes structure and its potential applications in tuning absorption frequencies for specific purposes.

**Figure 7:**
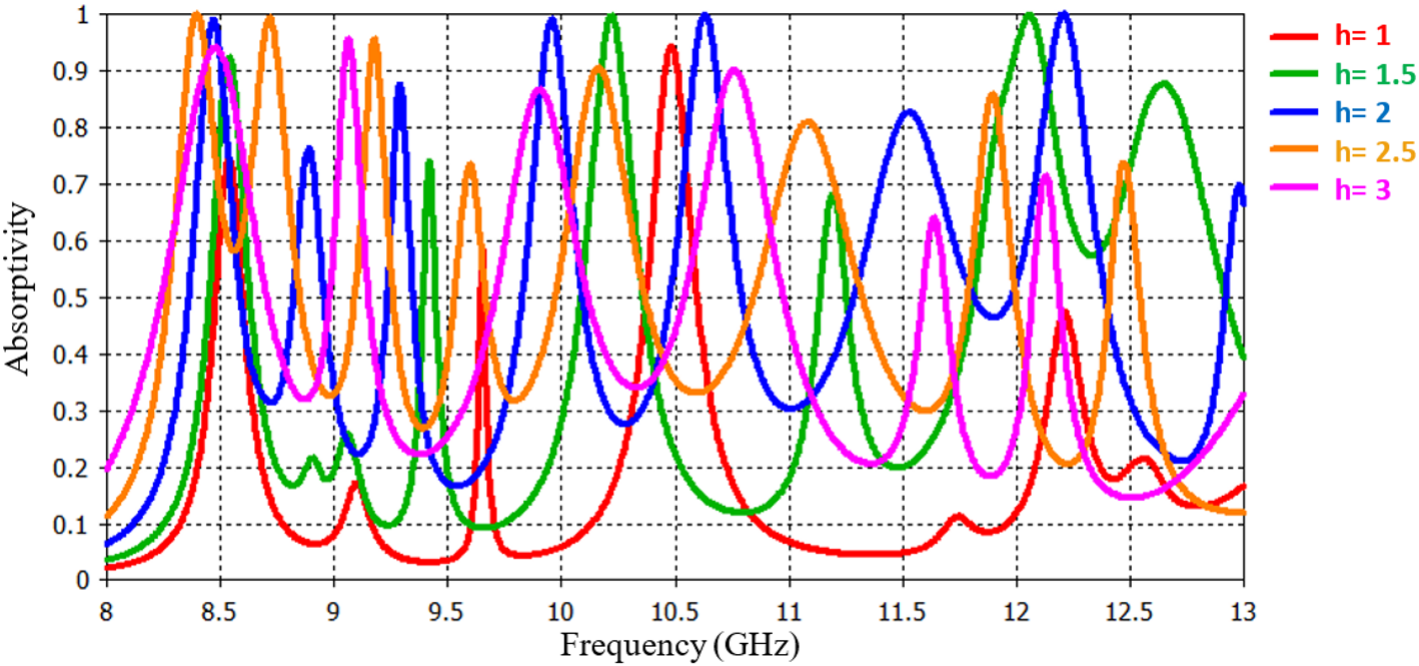
Absorption response for various thicknesses of the FR4 substrate, denoted as ‘h’.

The research also delves into the examination of how varying thicknesses of the FR4 substrate, denoted as ‘h’, can influence absorption performance. The investigation encompasses the consideration of five distinct substrate thickness values: 1 mm, 1.5 mm, 2 mm, 2.5 mm, and 3 mm. Illustrated in Fig. 7, the absorption characteristics, encompassing resonance frequencies and absorption coefficients, exhibit noteworthy fluctuations based on the chosen substrate thickness.

In contrast to the previous situation, there's a clear pattern: when the substrate gets thicker, resonance frequencies decrease, and when it gets thinner, resonance frequencies increase. Out of all the thickness values tested, 2 mm stands out as the best choice. It provides the highest absorption rates and meets the desired criteria perfectly. Therefore, the design opts for a 2 mm substrate thickness as the most suitable option.

### Sensing examination

**Figure 8:**
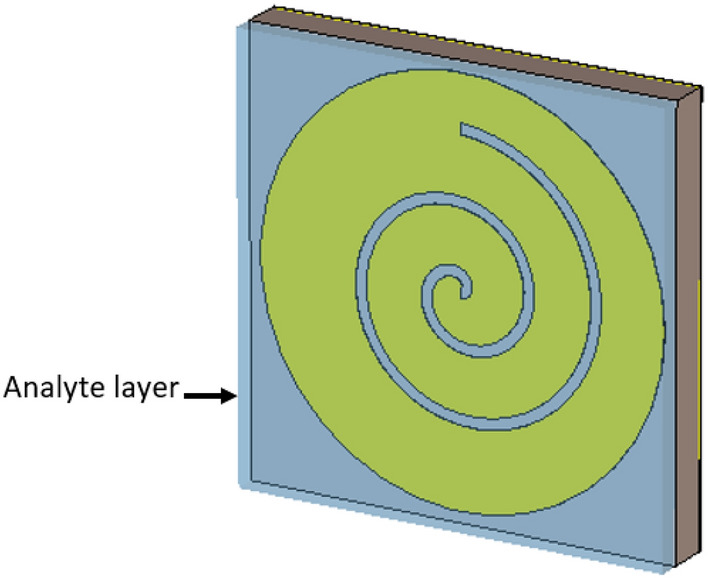
Sensor design (demonstrates the positioning of the analyte layer atop the layer of Archimedes loops).

The research investigates the sensing capabilities of a specific structure, as illustrated in Fig. 8. This examination involves the placement of a 1 mm analyte layer over the top of the Archimedes loops layer. The primary focus is on monitoring the resonant frequency shift resulting from the introduction of the analyte layer onto the patch. This frequency shift serves as a key indicator for assessing the sensing characteristics of the structure.

To gain deeper insights into the behavior of the material, a delve into the essential parameters, including the effective permittivity (ε_eff_), effective permeability (μ_eff_), ‘n_i_’, and wave impedance (z). These parameters play a pivotal role in characterizing the structure’s response to the analyte layer. They are calculated using various techniques, such as transmission-reflection (TR), Lossy-Drude, and Nicolson-Ross-Weir methods. Specifically, for Lossy-Drude and Nicolson-Ross-Weir techniques, ‘n’ is determined as follows: n = cos^–1^ ((1–S_11_+S_22_)/(2S_21_))/kδ, and ‘z’ is derived as: z = sqr[(1+S_11_)^2^–S_22_]/[(1–S_11_)^2^–S_22_].

Throughout this analysis, the analyte thickness remains constant at 1 mm. This deliberate choice allows us to assess the sensor’s effectiveness in detecting even the slightest changes. It’s worth noting that resonance shifts become inconsequential for analyte thicknesses below 0.5 mm, which reinforces our decision to set the minimum thickness at 1 mm.

**Figure 9:**
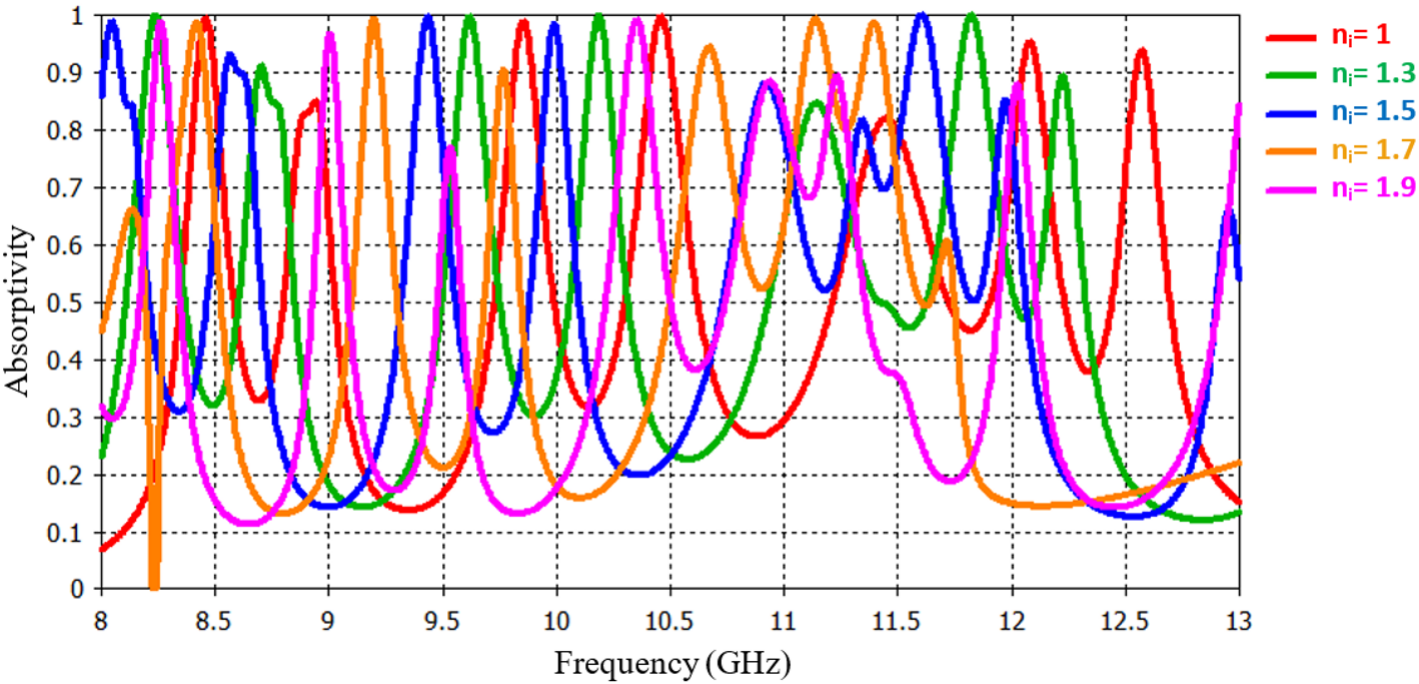
Absorption response for various refractive index.

The experimental results indicate that as we vary the refractive index (n_i_) values within the range of 1 to 1.9 see Fig. 9, a consistent trend emerges. Specifically, the resonant frequency shifts towards the lower frequency side, a behavior directly correlated with changes in the refractive index of the analyte. These findings provide valuable insights into the sensor’s sensitivity and its potential applications in detecting subtle variations in analyte properties.

Table 2 provides a comprehensive comparison between the innovative sensor inspired by metamaterial (MTM) and several other sensors documented in the literature. This comparison encompasses crucial parameters such as frequency band, Q-factor, and analyte layer thickness. In^2^, the sensor offers a frequency band of 2.4–4.2, a Q-factor of 30, and an analyte layer thickness of 3. However, in^3^, these values are 2–6, 60, and 1.6, respectively. Reference^4^ demonstrates an impressive Q-factor, although it is accompanied by a considerably thick analyte layer. A similar issue arises in^21^ and^22^. Notably, the proposed sensor exhibits an overall performance that surpasses that of the other sensors mentioned in the literature.

**Table 2:** Comparison the suggested design with other design in literature.

Ref	Operating frequency (GHz)	Max. Q-factor	Analyte layer thickness (mm)
^2^	2.4–4.2	30	3
^3^	2–6	60	1.6
^4^	8–12	135	10
^21^	8–12	NA	10
^22^	8–12	75	10
This work	8–13	84.5	1

## Conclusion

In conclusion, our study has successfully developed and simulated a planar complementary Archimedes-based metamaterial absorber, with a specific focus on its potential application in refractive index sensing. What sets our design apart is its simplicity, as it comprises just three layers: an FR4 dielectric substrate sandwiched between two copper layers. An important characteristic to highlight is its polarization-dependent absorption behavior, a result of the distinct resonance patterns observed in both the x and y directions. Furthermore, the resonator has explored the refractive index sensing potential of this structure by introducing a 1 mm analyte layer atop the patch structure. Our refractive index sensing analysis reveals a high-quality factor value of 84.5, highlighting the sensor's precision and sensitivity. Taken together, these findings position our absorber as a highly promising candidate for a wide range of microwave sensing applications, with its simplicity and exceptional performance making it a valuable asset in the field of metamaterial-based sensor technology.

## Data Availability

The datasets are available within the manuscript.
